# Subclinical myocardial changes in rheumatoid arthritis: cardiovascular magnetic resonance evidence of immuno-inflammatory remodeling

**DOI:** 10.3389/fcvm.2025.1607018

**Published:** 2025-08-21

**Authors:** Zoltan Tarjanyi, Liliana Szabo, Nikoletta Mong, Adil Mahmood, Zsofia Dohy, Zsofia Dora Drobni, Alexisz Panajotu, Laszlo Tothfalusi, Agnes Szappanos, Zahra Raisi-Estabragh, Bela Merkely, Gyorgy Nagy, Hajnalka Vago

**Affiliations:** ^1^ Heart and Vascular Centre, Semmelweis University, Budapest, Hungary; ^2^ William Harvey Research Institute, NIHR Barts Biomedical Research Centre, Queen Mary University of London, London, United Kingdom; ^3^ Barts Heart Centre, St Bartholomew’s Hospital, Barts Health NHS Trust, London, United Kingdom; ^4^ Department of Rheumatology and Immunology, Semmelweis University, Budapest, Hungary; ^5^ Faculty of Pharmacy, Department of Pharmacodynamics, Semmelweis University, Budapest, Hungary; ^6^ Department of Sports Medicine, Semmelweis University, Budapest, Hungary; ^7^ Department of Genetics, Cell- and Immunobiology, Semmelweis University, Budapest, Hungary

**Keywords:** rheumatoid arthritis, cardiovascular magnetic resonance, myocardial fibrosis, systemic inflammation, subclinical cardiac involvement

## Abstract

**Objectives:**

Rheumatoid arthritis (RA) is associated with increased cardiovascular (CV) risk, yet the mechanisms remain unclear. This study aimed to evaluate myocardial structure, function, and tissue characterization using cardiovascular magnetic resonance (CMR) in RA patients and explore associations with RA disease severity.

**Methods:**

This mixed case-control study included 48 RA patients and 34 age- and sex-matched controls. RA patients were enrolled based on ACR/EULAR criteria, excluding other autoimmune diseases or significant coronary artery calcification. CMR assessed myocardial structure, function, and tissue characteristics, including native T1/T2 mapping, ventricular volumes, strain analysis, and late gadolinium enhancement. Linear regression models adjusted for age, sex, hypertension, and diabetes evaluated associations between RA characteristics and CMR parameters.

**Results:**

RA patients exhibited elevated native T1 values (980 ± 34 ms vs. 955 ± 33 ms; *P* < 0.01), indicative of subclinical myocardial fibrosis. Left ventricular global longitudinal strain (GLS) was reduced (22 ± 2% vs. 24 ± 3%; *P* < 0.01), and increased left ventricular mass and remodeling were observed. Right ventricular end-diastolic and end-systolic volume indices were lower in RA patients (RVEDVi: 68 ± 14 ml/m^2^ vs. 75 ± 12 ml/m^2^, *P* = 0.02). Disease duration correlated negatively with GLS (*β* = −0.06, *P* < 0.05), while higher DAS28 scores were linked to reduced ejection fraction (*β* = −4.11, *P* < 0.05).

**Conclusions:**

This study demonstrates significant myocardial alterations in RA patients, including fibrosis, impaired systolic function, and ventricular remodeling, linked to disease severity. These findings highlight the need for early CV risk assessment and inflammation control to mitigate CV complications in RA.

## Graphical Abstract

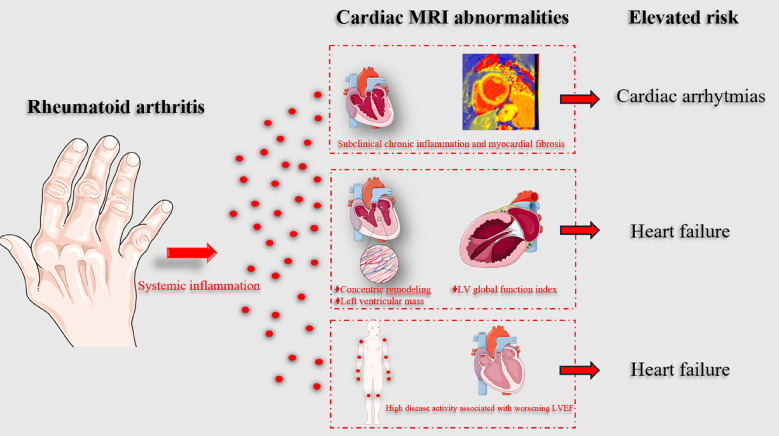

The figure illustrates the relationship between rheumatoid arthritis (RA), systemic inflammation, and its cardiovascular effects. Chronic inflammation in RA leads to myocardial fibrosis and concentric remodeling, as identified by cardiac MRI. These structural and functional abnormalities, including increased left ventricular mass and reduced global function, contribute to elevated risks of cardiac arrhythmias and heart failure. High RA disease activity further exacerbates left ventricular ejection fraction (LVEF) decline, emphasizing the importance of early cardiovascular assessment and intervention.

## Introduction

Rheumatoid arthritis (RA) is a chronic autoimmune disease characterized by joint inflammation, pain, and stiffness, affecting approximately 0.4%–0.6% of the population (1). The disease is characterized by periods of disease flares and remissions, and is frequently associated with comorbidities and extra-articular manifestations (2). The treat-to-target (T2T) concept can be considered as a fundamental therapeutic strategy of RA. Several essential elements of this therapeutic approach include an individual selection of a therapeutic target and determining the methods necessary to achieve it (3). Conventional synthetic, biological and targeted synthetic disease-modifying antirheumatic drugs (DMARDs) and glucocorticoids (GCs) are used to achieve the therapeutic goal of remission (4, 5). Despite adequate therapy, many patients remain symptomatic and are-, referred to as difficult-to-treat (D2T) (6). In addition, the exact determination of RA activity, the monitoring of response to treatment, and the prediction of extra-articular manifestations of the disease have not been resolved, despite the use of the disease activity score (DAS28), the rheumatoid factor (RF) and the anti-citrulined protein antibody (ACPA) (7, 8).

In addition to synovial joint involvement RA is associated with a significantly increased risk of cardiovascular (CV) diseases (9–12), imposing a substantial effect on patient's health (13). CV morbidity and mortality with ischaemic heart disease (IHD) and congestive heart failure (CHF) are higher in RA patients compared to the general population, with an increased CV mortality risk of up to 50% (14–16). The increased CV risk is attributed to a synergy between traditional CV risk factors and RA-related factors such as persistent systemic inflammation, metabolic disturbances, and treatment-related adverse effects (17, 18). Chronic systemic inflammation can lead to accelerated atherosclerosis, endothelial dysfunction, and plaque instability, affecting atherogenesis both directly and indirectly, thus increasing the risk of acute coronary events and left ventricular dysfunction (19, 20). Furthermore, RA patients have a 2-fold higher risk of sudden cardiac death (SCD) compared to non-RA patients, suggesting an increase in the frequency of malignant ventricular arrhythmias. In addition to accelerating the development of IHD and CHF, chronic systemic inflammation increases the development of arrhythmias by directly modifying the heart's electrophysiological homeostasis through cytokines (IL-1, IL-6, TNFα) (21).

The precise mechanisms underlying the development of arrhythmogenic substrates in RA patients are not fully understood. Structural changes in the heart associated with IHD and CHF may play a crucial role in elevating the arrhythmic risk in RA patients, promoting the development of conditions that predispose them to life-threatening arrhythmias.

Cardiovascular magnetic resonance (CMR) imaging is a non-invasive diagnostic tool that uniquely evaluates multiple aspects of the heart, including myocardial structure, function, tissue characterization, perfusion, and inflammation (13, 14). Therefore CMR imaging provides an excellent diagnostic tool to investigate the CV effects of RA comprehensively. Previous studies using CMR to assess the CV consequences of RA have demonstrated subclinical changes in myocardial structure (22, 23). These include diffuse myocardial fibrosis, characterized by increased extracellular volume and T1 mapping abnormalities, indicating extensive myocardial scarring. The cytokine release - subclinical inflammation - subclinical myocardial fibrosis axis relationship with the disruption of the local myocardial electrophysiological balance may play a significant role in the two-fold increased risk of SCD linked to RA. Consequently, it highlights the importance of CMR in the detection of early cardiac subclinical fibrosis.

We aimed to evaluate CMR-based measures of myocardial structure, function, and tissue characterization, and to explore the relationships between clinical indicators of disease severity and CMR parameters in patients with RA, alongside a healthy control population without significant CV history.

## Methods

### Study population

We performed a mixed case-control study, where patients with RA were prospectively enrolled and controls were retrospectively selected. RA patients were prospectively enrolled from two outpatient clinics (Rheumatology Department at Semmelweis University and the National Institute of Locomotor Diseases and Disabilities, Budapest, Hungary). The sample size was determined based on comparable studies reported in the literature (24–26). Inclusion criteria for the patients included the following: aged 18 years or older, ACPA positive, diagnosed with RA based on the 2010 American College of Rheumatology/European League Against Rheumatism classification criteria (27) at least five years ago. We excluded all individuals with concurrent autoimmune diseases, known ischemic heart disease, history of malignant diseases, chronic infections (with or without fever), or known claustrophobia. Moreover, as detailed below, a non-contrast enhanced CT was performed to assess coronary calcification, and those with an Agatston Ca-score >500 were excluded from the study. Control population was retrospectively selected from a cohort of verified healthy volunteers at Semmelweis University Heart and Vascular Center who had CMR data available. Controls were age and sex matched to cases.

Data on patient medical history, CV risk factors, including treated hypertension, diabetes and current smoking (smoker/non-smoker) was collected using electronic health records. Medications were recorded including GCs, NSAIDs, conventional and targeted DMARDs, beta-blockers, renin-angiotensin-aldosterone system blockers and statins. Blood markers of systemic inflammation, such as, C Reactive Protein and white cell count were measured at enrolment. Disease activity was evaluated by using the 28-joint (DAS28) score.

### Patient involvement

Patients were not directly involved in the design, recruitment, or conduct of this study. All participants provided written informed consent before enrollment, and the study was conducted in accordance with the Declaration of Helsinki. Patient data were anonymized, and ethical approval was obtained from national and institutional ethics committees (approval number: IF 567-4-2016).

Although patients did not contribute to the study design, the findings have significant implications for patient care. By excluding individuals with known coronary artery disease, this study uniquely focused on the direct cardiovascular effects of rheumatoid arthritis, offering insights that may improve risk assessment and management strategies for RA patients. The results will be disseminated through scientific publications and clinical presentations, ensuring that both healthcare professionals and patient communities benefit from the findings.

### Calcium scoring

All RA participants underwent non–contrast-enhanced, prospectively ECG-triggered scan of the heart using a dedicated multidetector cardiac CT scanner (CardioGraphe, GE Healthcare, Chicago, IL, USA) at the Heart and Vascular Center of Semmelweis University. The images were acquired in cranio-caudal direction during breath hold in inspiration, at 78% of the R-R interval, with a slice thickness of 3.0 mm. The following acquisition parameters were used: 140 × 0.48 mm detector collimation, 240 ms gantry rotation time, 120 kV tube voltage, and 30 mAs tube current. The quantification of coronary artery calcium (CAC) was performed on the axial images on a per-patient and per-vessel basis using a semi-automatic software (Heartbeat-CS, Philips Healthcare, Best, The Netherlands). Coronary artery calcium score (CCS) was computed by the standard calcium scoring algorithm according to Agatston.

### CMR protocol

CMR examinations were conducted on a 1.5 T MR scanner (Magnetom Aera; Siemens Healthcare, Erlangen, Germany). The protocol included balanced steady state free precession (b-SSFP) cine sequencescine movies, T2-weighted spectral presaturation with inversion recovery, T2 mapping using T2-prep balanced steady-state free precession (b-SSFP), and T1 mapping using both long-T1 5(3)3 and short-T1 5(3)3 modified look-locker inversion recovery. Stress adenosine perfusion and late gadolinium enhancement (LGE) imaging were also performed.

Standard cine slices were acquired in the four-chamber, two-chamber, and three-chamber long-axis views, along with a short-axis stack covering both ventricles from base to apex. Pharmacological stress perfusion was conducted using 140 µg/kg/min adenosine for 3 min a 0.15 mmol/kg gadolinium-based contrast bolus (Gadobutrol; Gadovist, Bayer-Schering Pharma) at a rate of 2–3 ml/s. The ’splenic switch-off' sign was used to confirm adequate hyperemia. LGE images were acquired using a segmented inversion recovery sequence 10–15 min after contrast administration.

Healthy controls underwent a non-contrast CMR scan, including cine movies, T2-weighted spectral presaturation with inversion recovery, T2 mapping using T2-prep balanced steady-state free precession (b-SSFP), and T1 mapping using both long-T1 5(3)3 and short-T1 5(3)3 modified look-locker inversion recovery.

### CMR post-processing

Post-processing analyses utilized Medis Suite Software (Medis Medical Imaging Software, The Netherlands). Automated artificial intelligence-based contour detection was employed to determine left ventricular (LV) and right ventricular (RV) volumes, function, and mass from the short-axis stack, with manual corrections made as needed. We considered the following metrics: end-diastolic volume (LVEDV, RVEDV), end-systolic volume (LVESV, RVESV), stroke volume (LVSV, RVSV), LV mass (LVM), LV end-diastolic wall thickness (EDWT), ejection fraction (LVEF, RVEF). The LV mass-to-volume ratio was calculated using LVM/LVEDV. Left ventricular global function index (LVGFI) integrates left ventricular structure and global function into a single index. The left ventricular global function index (LVGFI) is calculated as [(LV stroke volume/LV global volume) × 100], where LV myocardial volume is derived by dividing LV myocardial mass by the myocardial density (1.05 g/ml) (28). T1 and T2 myocardial native relaxation times were measured in the midventricular or basal septum of the myocardium using motion-corrected images (29). Quantitative deformation analysis was performed on cine movies using feature-tracking strain analysis, extracting LV global longitudinal (GLS) and circumferential (GCS) strain. For global dyssynchrony measurement, mechanical dispersion was calculated as the standard deviation of the time-to-peak longitudinal strain and expressed as a percentage of the cardiac cycle. Myocardial deformation was assessed pre- and post-adenosine administration on cine images to understand how the contraction pattern changes due to stress. Global longitudinal and circumferential strain values are reported as their absolute values for consistency and improved interpretation (30). Stress perfusion defects were evaluated visually, and the criteria for perfusion defect definition were established according to the SCMR guideline (SCMR ref given before). LGE interpretation was conducted visually by two observers, who assessed the presence and pattern of myocardial LGE. In the event of a disagreement between the two observers, a third opinion was sought from a CMR specialist with Level 3 certification from the European Association of Cardiovascular Imaging to reach a consensus. Non-ischaemic LGE was defined as midmyocardial and/or subepicardial myocardial LGE, confirmed in two perpendicular views. An ischaemic LGE pattern was identified as subendocardial to transmural.

### Statistical analysis

Data were analysed using R [R Core Team (2022). R: A language and environment for statistical computing. R Foundation for Statistical Computing, Vienna, Austria. URL https://www.R-project.org/], tables and figures were prepared with flextable [Gohel D, Skintzos P (2024). _flextable: Functions for Tabular Reporting_. R package version 0.9.7, https://CRAN.R-project.org/package=flextable] and ggplot2 (H. Wickham. ggplot2: Elegant Graphics for Data Analysis. Springer-Verlag New York, 2016.). We used R-Studio (Posit Software, USA) for the analysis. We tested the normality of continuous variables using the Shapiro–Wilk test and QQ plots. Continuous variables were summarised using means and standard deviations or median and interquartile ranges and categorical variables as counts and percentages. To compare CMR parameters between patients with RA and healthy controls, we used independent sample *t*-tests. We employed linear regression analyses to investigate the association of RA with CMR parameters. In these models, each CMR parameter served as the dependent variable, and RA was the independent variable, with healthy volunteers used as the reference category. Initially, we constructed unadjusted models (Model 1), followed by models adjusted for age and, sex (Model 2), and finally we reported the results from models adjusted for age, sex, hypertension, and diabetes (Model 3). We reported results as regression coefficients with their respective 95% confidence intervals (CIs). To enhance interpretation, we also report results from Model 3 as standardized regression coefficients with 95% confidence intervals. In the subset of patients with RA, we used multiple linear regression models to explore the association between clinical disease characteristics (disease duration, DAS28, and ACPA) and CMR parameters. These models were adjusted for age, sex, hypertension, and diabetes, and results were reported as regression coefficients with 95% CIs. Finally, we assessed deformation changes in response to adenosine infusion using paired sample *t*-tests. All statistical tests were two-tailed, and a *p*-value of less than 0.05 was considered statistically significant.

## Results

### Baseline characteristics

A total of 82 individuals underwent CMR imaging, comprising 48 patients with RA and 34 healthy volunteers (Table 1). The mean age of patients with RA was 58 years with a greater proportion of females (77%). The median disease duration was 19.0 years. Patients had moderate disease activity (median DAS28 score 3.8 units) with a median ACPA concentration of 497 units, and 67% were on biologic therapy. Hypertension and diabetes mellitus were present in 44% and 8% of patients, respectively, and 25% were smokers.

**Table 1:** Baseline characteristics.

Variable	RA patient (*n* = 48)	Healthy volunteer (*n* = 34)
Age, years	58 ± 10	55 ± 5
Sex, *n* (%)	Female: 37 (77%); Male: 11 (23%)	Female: 22 (65%); Male: 12 (35%)
Disease duration, years	19 ± 12	NA
DAS28 (metrics)	3.8 ± 1.5	NA
ACPA (metrics)	497 (260, 1,600)	NA
Ca score (metrics)	0.5 (0, 75)	NA
Biological therapy, *n* (%)	32 (67%)	NA
Smoking, *n* (%)	12 (25%)	0 (0%)
Myocardial infarction, *n* (%)	0 (0%)	0 (0%)
Stroke, *n* (%)	3 (6%)	0 (0%)
Hypertension, *n* (%)	21 (44%)	0 (0%)
Diabetes mellitus, *n* (%)	4 (8%)	0 (0%)
ACEi, *n* (%)	12 (25%)	NA
Beta blocker, *n* (%)	13 (27%)	NA
Statin, *n* (%)	2 (4%)	NA

Comprehensive comparison of baseline characteristics between RA patients and healthy volunteers. Continuous variables are given as mean and standard deviation for values showing normal distribution and median (IQR) for thos showing non-normal distribution. Categorical variables are reported as *n* (%).

RA, rheumatoid arthritis, DAS28, disease activity score-28; ACPA, anti-citrullinated protein antibody; Ca score, coronary artery calcium score; NA, not applicable; ACEi, angiotensin-converting enzyme inhibitor.

### Comparison of CMR values between RA patients and controls

CMR indices of LV size, left ventricular end-diastolic volume index (LVEDVi) and left ventricular end-systolic volume index (LVESVi), did not differ between RA patients and controls (Table 2). Left ventricular ejection fraction (LVEF) was similar between the two groups. In contrast, LV GLS showed significantly lower longitudinal function than healthy controls (GLS: 22 ± 2 vs. 24 ± 3%; *P* < 0.01). Native T1 values were significantly higher in RA (Native T1 septal ROI: 980 ± 34 ms) in comparison to controls (955 ± 33 ms; *P* < 0.01). Conversely, the two groups had no significant differences in T2 values.

**Table 2:** Comparison of CMR metrics between RA patients and healthy volunteers.

Variable	RA patients	Healthy volunteers	*P*-value
LVEDVi (ml/m^2^)	73 ± 15	76 ± 11	0.21
LVESVi (ml/m^2^)	28 ± 7	28 ± 6	0.90
LVSVi (ml/m^2^)	45 ± 9	48 ± 8	0.10
LVEF (%)	62 ± 5	63 ± 5	0.46
LVGFI (%)	50 ± 5	53 ± 6	**0.014**
LVMi (g/m^2^)	42.8 ± 8.7	41 ± 5.4	0.24
LVM/LVEDV ratio	0.6 ± 0.1	0.5 ± 0	**<0.001**
T1 septal (ms)	979 ± 33	955 ± 33	**<0.001**
T1 lateral (ms)	973 ± 39	957 ± 27	**0.02**
T2 septal (ms)	46 ± 3	46 ± 2	0.86
T2 lateral (ms)	47 ± 3	47 ± 3	0.79
GLS (%)	22 ± 2	24 ± 3	0.01
GCS (%)	34 ± 4	35 ± 4	0.389
Mechanical dispersion	13 ± 3	11 ± 4	**0.015**
RVEDVi (ml/m^2^)	68 ± 14	75 ± 12	**0.030**
RVESVi (ml/m^2^)	25 ± 8	28 ± 7	0.069
RVSVi (ml/m^2^)	44 ± 8	47 ± 8	0.058
RVEF (%)	64 ± 6	63 ± 5	0.380

A comparison of CMR metrics between RA patients and healthy volunteers.

RA, rheumatoid arthritis; LVEDVi, left ventricular end-diastolic volume index; LVESVi, left ventricular end-systolic volume index; LVSVi, left ventricular stroke volume index; LVEF, left ventricular ejection fractionLVGFI, left ventricular global function index; LVMi, left ventricular mass index; LVM/LVEDV ratio, left ventricular mass-to-end-diastolic volume ratio; T1 septal/lateral, native T1 mapping values for septal and lateral myocardial segments; T2 septal/lateral, native T2 mapping values for septal and lateral myocardial segments; GLS, global longitudinal strain; GCS, global circumferential strain; Mechanical dispersion, standard deviation of time-to-peak strain as a measure of dyssynchrony; RVEDVi, right ventricular end-diastolic volume index; RVESVi, right ventricular end-systolic volume index; RVSVi, right ventricular stroke volume index; RVEF, right ventricular ejection fraction.

Bold values indicate statistically significant differences or associations (*P* < 0.05).

RA patients demonstrated lower right ventricular end-diastolic volume index (RVEDVi) (68 ± 14 ml/m^2^) than healthy volunteers (75 ± 12 ml/m^2^; *P* = 0.02). Similarly, patients with RA had lower right ventricular end-systolic volume index (RVESVi) (25 ± 8 ml/m^2^) in comparison to controls (28 ± 7 ml/m^2^; *P* = 0.05). The two groups had no significant differences in CMR indices of RV systolic function (RVEF and RVSVi).

### Association between RA and CMR values

In univariate linear regression models, RA was associated with markers of worse LV function: decreased left ventricular global function index (LVGFI) and GLS. Having RA disease was also associated with an increased mechanical dispersion suggestive of increased dyssynchrony of LV contraction, higher LV concentricity, and increased T1 values: T1 (septal) [23 (8–38)] (Table 3). RA also showed a significant negative association with RVEDVi and RVESVi.

**Table 3:** Association between RA exposure and CMR values in the whole cohort.

Variable	Model 1 (CI 95%)	*P*-value	Model 2 (CI 95%)	*P*-value	Model 3 (CI 95%)	*P*-value
LVEDVi (ml/m^2^)	−3.23 (−9.02–2.56)	0.27	−0.51 (−5.7–4.68)	0.85	1.55 (−4.19–7.29)	0.59
LVESVi (ml/m^2^)	0.07 (−2.83–2.97)	0.96	1.4 (−1.2–4)	0.29	2.28 (−0.64–5.2)	0.12
LVSVi (ml/m^2^)	−3.07 (−6.94–0.8)	0.12	−1.79 (−5.53–1.95)	0.34	−0.57 (−4.73–3.59)	0.78
LVEF (%)	−1.07 (−3.24–1.1)	0.33	−1.6 (−3.74–0.54)	0.14	−1.09 (−3.52–1.34)	0.38
LVGFI (%)	−0.03 (−0.06–0)	**0.01**	−0.04 (−0.06–0.02)	**<0.001**	−0.03 (−0.06–0)	**0.04**
LVMi (g/m^2^)	2.03 (−1.32–5.38)	0.23	3.58 (0.58–6.58)	**0.02**	3.86 (0.51–7.21)	**0.02**
LVM/LVEDV ratio	0.05 (0.02–0.08)	**<0.001**	0.05 (0.02–0.08)	**<0.001**	0.04 (0–0.08)	**0.03**
T1 septal (ms)	23.14 (8.06–38.22)	**<0.001**	21.53 (8.31–34.75)	**<0.001**	24.86 (10.11–39.61)	**<0.001**
T1 lateral (ms)	16.67 (1.09–32.25)	**0.04**	17.54 (3.24–31.84)	**0.02**	20.11 (3.96–36.26)	**0.02**
T2 septal (ms)	0.24 (−0.92–1.4)	0.68	0.06 (−1.1–1.22)	0.91	0.14 (−1.18–1.46)	0.83
T2 lateral (ms)	0.32 (−1.05–1.69)	0.65	0.1 (−1.2–1.4)	0.88	0.81 (−0.63–2.25)	0.27
GLS (%)	−1.56 (−2.61–0.51)	**<0.001**	−1.59 (−2.67–0.51)	**<0.001**	−1.04 (−2.21–0.13)	0.08
GCS (%)	−0.79 (−2.59–1.01)	0.39	−1.22 (−3–0.56)	0.17	−1.13 (−3.13–0.87)	0.27
Mechanical dispersion	2.11 (0.47–3.75)	**0.01**	2.02 (0.36–3.68)	**0.02**	1.35 (−0.5–3.2)	0.15
RVEDVi (ml/m^2^)	−6.43 (−12.33–0.53)	**0.03**	−3.62 (−8.82–1.58)	0.17	−0.81 (−6.46–4.84)	0.77
RVESVi (ml/m^2^)	−3.08 (−6.45–0.29)	0.07	−1.52 (−4.44–1.4)	0.31	−0.07 (−3.34–3.2)	0.97
RVSVi (ml/m^2^)	−3.47 (−7.08–0.14)	0.06	−2.22 (−5.7–1.26)	0.21	−0.82 (−4.59–2.95)	0.67
RVEF (%)	1.1 (−1.45–3.65)	0.39	0.41 (−2.03–2.85)	0.74	−0.09 (−2.86–2.68)	0.95

Linear regression models assess the association between RA and CMR values in the entire cohort. Model 1 reports unadjusted associations, Model 2 is adjusted for age and sex, finally Model 3 reports results adjusted for age, sex, hypertension and diabetes. Raw regression coefficients with their corresponding 95% confidence intervals are reported with their corresponding *P*-value.

RA, rheumatoid arthritis; LVEDVi, left ventricular end-diastolic volume index; LVESVi, left ventricular end-systolic volume index; LVSVi, left ventricular stroke volume index; LVEF, left ventricular ejection fraction; LVGFI, left ventricular global function index; LVMi, left ventricular mass index LVM/LVEDV ratio, Left ventricular mass-to-end-diastolic volume ratio; T1 septal/lateral, native T1 mapping values for septal and lateral myocardial segments; T2 septal/lateral, native T2 mapping values for septal and lateral myocardial segments; GLS, global longitudinal strain; GCS, global circumferential strain; Mechanical dispersion, standard deviation of time-to-peak strain as a measure of dyssynchrony; RVEDVi, right ventricular end-diastolic volume index; RVESVi, right ventricular end-systolic volume index; RVSVi, right ventricular stroke volume index; RVEF, right ventricular ejection fraction.

Bold values indicate statistically significant differences or associations (*P* < 0.05).

In models adjusted for age and sex, RA showed a strong positive association with measures of LV adverse remodelling including LVMi [3.58 (0.58–6.58)], LVM/LVEDV ratio, mechanical dispersion and septal T1 [21.53 (8.31–34.75)] values whereas it was negatively associated with sensitive measures of cardiac function including LVGFI and-, GLS [−1.59 (−2.67 to −0.51)].

**Figure 1:**
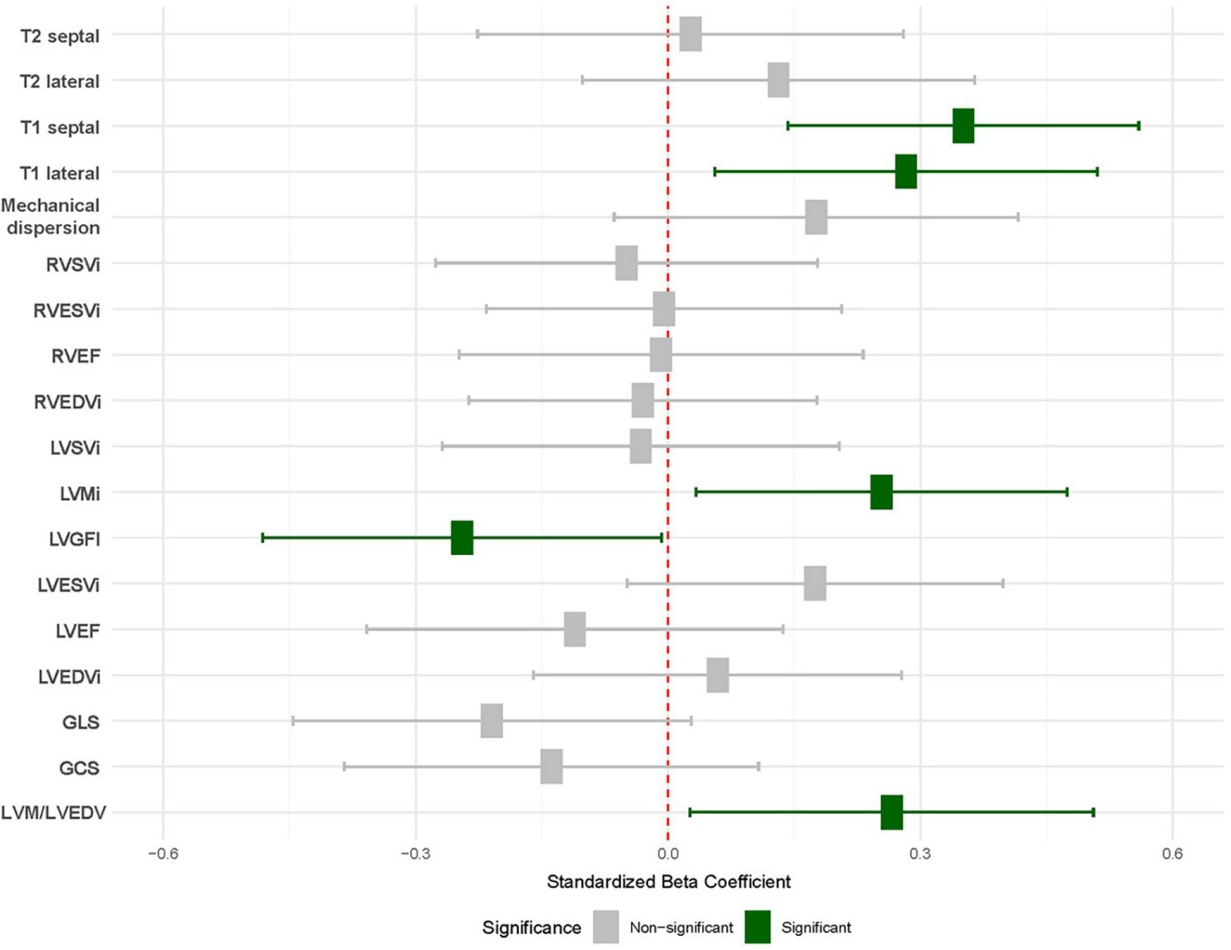
Association between RA and CMR values. Results from linear regression models adjusted age, sex, hypertension and diabetes. Each row corresponds with an individual model. Significant results are shown in green. Baseline was defined as healthy volunteers (red). T2 septal/lateral, native T2 mapping values for the septal and lateral myocardial segments; T1 septal/lateral, Native T1 mapping values for the septal and lateral myocardial segments; Mechanical dispersion, Standard deviation of time-to-peak strain as a measure of ventricular dyssynchrony; RVSVi, right ventricular stroke volume index; RVESVi, right ventricular end-systolic volume index; RVEF, right ventricular ejection fraction; RVEDVi, right ventricular end-diastolic volume index; LVSVi, left ventricular stroke volume index; LVMi, left ventricular mass index; LVGFI, left ventricular global function index; LVESVi, left ventricular end-systolic volume index; LVEF, left ventricular ejection fraction; LVEDVi, left ventricular end-diastolic volume index; GLS, global longitudinal strain; GCS, global circumferential strain; LVM/LVEDV, left ventricular mass-to-end-diastolic volume ratio.

Finally, in models adjusted for age, sex, hypertension, and diabetes, the associations observed between RA and LVGFI [−0.03 (−0.06–0)], LVM/LVEDV ratio [0.04 (0–0.08)], and T1 values [24.86 (10.11, 39.61)] and LVMi [3.86 (0.51–7.21)] remained significant (Figure 1, Table 3).

### Association between clinical parameters and CMR values in RA patients

In linear regression models adjusted for age, sex, hypertension and diabetes, of the clinical parameters considered in our study (disease duration, DAS28). RA disease duration showed a negative association with GLS [−0.06 (−0.11, −0.01), *p* < 0.05], and increasing DAS28 showed a significant negative association with LVEF [−4.11 (−7.89, −0.34) *p* < 0.05]. Other CMR parameters showed no significant associations with disease duration or DAS28 score (Table 4).

**Table 4:** Association between clinical characteristics and CMR measures of RA patients.

RA disease characteristics	CMR variables	Coefficient (95 CI)	*P*-value
Disease length	LVEDVi (ml/m^2^)	0.08 (−0.26, 0.41)	0.642
Disease length	LVESVi (ml/m^2^)	−0.03 (−0.2, 0.13)	0.705
Disease length	LVSVi (ml/m^2^)	0.12 (−0.1, 0.34)	0.263
Disease length	LVEF (%)	0.03 (−0.1, 0.16)	0.619
Disease length	LVGFI (%)	0 (0, 0)	0.519
Disease length	LVMi (g/m^2^)	−0.02 (−0.24, 0.2)	0.855
Disease length	LVM/LVEDV ratio	0 (0, 0)	0.485
Disease length	RVEDVi (ml/m^2^)	0.05 (−0.24, 0.34)	0.725
Disease length	RVESVi (ml/m^2^)	0.07 (−0.11, 0.25)	0.418
Disease length	RVSVi (ml/m^2^)	−0.02 (−0.22, 0.17)	0.796
Disease length	RVEF (%)	−0.06 (−0.23, 0.11)	0.462
Disease length	T1 septal (ms)	0.08 (−0.79, 0.94)	0.859
Disease length	T1 lateral (ms)	0.12 (−0.92, 1.17)	0.813
Disease length	T2 septal (ms)	0.04 (−0.03, 0.12)	0.278
Disease length	T2 lateral (ms)	0 (−0.08, 0.08)	0.998
Disease length	ECV septal (%)	−0.11 (−0.27, 0.05)	0.168
Disease length	ECV lateral (%)	−0.08 (−0.27, 0.1)	0.359
Disease length	GLS (%)	−0.06 (−0.11, −0.01)	**0.046**
Disease length	GCS (%)	−0.09 (−0.19, 0.01)	0.084
Disease length	Mechanical dispersion	0.02 (−0.07, 0.12)	0.625
DAS28	LVEDVi (ml/m^2^)	4.05 (−6.12, 14.23)	0.426
DAS28	LVESVi (ml/m^2^)	3.61 (−1.29, 8.51)	0.145
DAS28	LVSVi (ml/m^2^)	−0.8 (−7.6, 6.01)	0.814
DAS28	LVEF (%)	−4.11 (−7.89, −0.34)	**0.034**
DAS28	LVGFI (%)	−0.04 (−0.08, 0)	0.078
DAS28	LVMi (g/m^2^)	1.4 (−5.21, 8)	0.672
DAS28	LVM/LVEDV ratio	−0.02 (−0.09, 0.05)	0.573
DAS28	RVEDVi (ml/m^2^)	0.35 (−8.48, 9.17)	0.937
DAS28	RVESVi (ml/m^2^)	3.08 (−2.35, 8.51)	0.259
DAS28	RVSVi (ml/m^2^)	−2.73 (−8.57, 3.11)	0.351
DAS28	RVEF (%)	−3.32 (−8.45, 1.82)	0.2
DAS28	T1 septal (ms)	9.3 (−16.95, 35.54)	0.478
DAS28	T1 lateral (ms)	−2.48 (−34.4, 29.45)	0.876
DAS28	T2 septal (ms)	1.34 (−1, 3.67)	0.255
DAS28	T2 lateral (ms)	−0.27 (−2.68, 2.14)	0.823
DAS28	ECV septal (%)	−2.83 (−7.42, 1.77)	0.22
DAS28	ECV lateral (%)	−2.56 (−7.81, 2.7)	0.33
DAS28	GLS (%)	−0.79 (−2.55, 0.97)	0.372
DAS28	GCS (%)	−1.95 (−5.17, 1.27)	0.229
DAS28	Mechanical dispersion	−1.48 (−4.37, 1.41)	0.308

Linear regression models, adjusted for age, sex, hypertension and diabetes. Raw regression coefficients and their corresponding 95% confidence interval is reported. DAS 28 values showed non-normal distribution and were log-transformed before we enter them into the linear regression model.

RA, rheumatoid arthritis; CMR, cardiovascular magnetic resonance; LVEDVi, left ventricular end-diastolic volume index; LVESVi, left ventricular end-systolic volume index; LVSVi, left ventricular stroke volume index; LVEF, left ventricular ejection fraction; LVGFI, left ventricular global function index; LVMi, left ventricular mass index; LVM/LVEDV ratio, Left ventricular mass-to-end-diastolic volume ratio; RVEDVi, right ventricular end-diastolic volume index; RVESVi, right ventricular end-systolic volume index; RVSVi, right ventricular stroke volume index; RVEF, right ventricular ejection fraction; T1 septal/lateral, native T1 mapping values for septal and lateral myocardial segments; T2 septal/lateral, native T2 mapping values for septal and lateral myocardial segments; ECV septal/lateral, extracellular volume fraction for septal and lateral myocardial segments; GLS, global longitudinal strain; GCS, global circumferential strain; Mechanical dispersion, standard deviation of time-to-peak strain as a measure of ventricular dyssynchrony; DAS28, disease activity score-28.

Bold values indicate statistically significant differences or associations (*P* < 0.05).

### CMR stress perfusion

Of the 41 patients who underwent CMR stress perfusion imaging, we did not detect inducible alterations. We compared measures of LV deformation pre- and post-adenosine stress perfusion, which showed increasing LV longitudinal and circumferential strain on exertion (Supplementary Figure S1).

### Late gadolinium enhancement

We observed late gadolinium enhancement (LGE) in five cases with non-ischaemic patterns. Three cases showed non-specific pattern with unknown clinical significance, one patient presented with LGE localized in the aortic annulus, and one patient with midmyocardial LGE in the basal anteroseptal segment resembling calcification.

## Discussion

RA is a systemic autoimmune disease characterized by symmetrical inflammatory polyarthritis. The most significant extra-articular manifestations include an increased risk of interstitial lung disease, central nervous system involvement, and heightened cardiovascular risk. RA was associated with a 48% increased risk of cardiovascular events and 50% higher with the mortality of cardiovascular disease compared to the healthy control subject (15, 16). The elevated cardiovascular disease risk is attributed to the chronic inflammatory state inherent to RA, which, in parallel with accelerated atherosclerosis, affects myocardial remodelling, leading to a heightened risk of heart failure, myocardial infarction, sudden cardiac death, and stroke (31). Endothelial dysfunction of RA is connected through many mechanisms to the inflammatory processes of the disease. In addition, chronic systemic inflammation increases the development of arrhythmias by directly modifying the heart's electrophysiological homeostasis through different cytokines (21).

Patients with RA face a two-fold increased risk of experiencing SCD compared to the general population. SCD is strongly associated with myocardial fibrosis, a process where excessive fibrous connective tissue accumulates in the heart. This fibrosis disrupts the normal electrical conduction in the heart, creating an arrhythmogenic substrate ultimately increasing the risk of SCD (32). In our study, we demonstrated that T1 values, which may indicate tissue changes such as increased myocardial inflammation or fibrosis, were significantly higher in patients with RA compared to healthy control individuals.

Heart failure is a major public health concern affecting nearly 63 million people worldwide (33, 34). Despite notable advancements in treatment and preventive measures, both mortality and morbidity rates remain elevated, and patients continue to experience a reduced quality of life. Patients with RA exhibit a higher risk of developing heart failure compared to individuals without RA, even after accounting for traditional CV risk factors and the presence of coronary artery disease (35). While the increased risk has been well established through previous studies, the precise structural tissue changes underlying this phenomenon have not been fully elucidated. In our research, we demonstrated that patients with RA have significantly elevated values of concentric remodeling, increased left ventricular (LV) mass, and decreased left ventricular global function index compared to healthy controls. Changes in these parameters have been previously associated with a heightened risk of developing heart failure in subsequent studies, highlighting the prognostic significance of these alterations (36–39). Furthermore, our analysis revealed a significant correlation between the DAS28 score and the decline in LVEF among patients with RA. DAS28 is a formula based on a mathematical calculation that includes the number of the most frequently affected 28 tender and swollen joints, self-assessment of health status using a visual analogue scale (VAS), and the value of acute phase response as erythrocyte sedimentation rate (ESR) or C-reactive protein (CRP). The association between higher disease activity and reduced systolic function suggests that persistent inflammation may exacerbate myocardial injury, thereby contributing to progression towards heart failure.

From a clinical standpoint, these findings hold significant implications. Our study demonstrates that RA patients present with higher myocardial T1 values, increased concentric remodeling, greater LV mass, and a reduced left ventricular global function index when compared to healthy controls, independent of confounding factors such as age, sex, and conventional CV risk factors. These results are pivotal in understanding the underlying mechanisms contributing to the elevated CV risk observed in RA patients. They underscore the importance of early CV risk assessment and management in RA patients. This could lead to more targeted therapies aimed not only at controlling joint inflammation but also at preventing or mitigating cardiac involvement. In our study, consistent with the international literature, we also demonstrated that worse DAS28 scores indicate the need for vigilant cardiac monitoring in patients with high disease activity RA.

When comparing our results with previous RA-CMR studies, some investigations have shown no significant changes in T1 times among RA patients, while others, consistent with our findings, have reported notable alterations in T1 values. The divergence between these results warrants further investigation. However, it can already be observed that studies involving RA patients with low to moderate disease activity, as indicated by DAS28-CRP scores, tend to show no significant changes in T1 values. In contrast, studies focusing on patients with moderate-high disease activity, such as ours, have demonstrated significant changes in T1 times (24–26). The inconsistency observed in T1 values among previous RA-CMR studies may also arise from differences in the methodology. In our current study, we aimed to more effectively exclude RA patients with potential ischemic heart disease by incorporating coronary calcium score assessment. Previous RA-CMR studies considered patients to be CAD negative if their medical history did not suggest ischemic heart disease. Consequently, the T1 alterations observed in our study were able to more specifically highlight the cardiac effects of chronic generalized inflammatory activity. Our cohort of patients with long-standing RA showed elevated T1 values, but no significant changes in T2 values. This is consistent with chronic low-grade inflammation and fibrotic remodeling rather than acute myocardial oedema. Prior CMR studies in early RA have sometimes reported elevated T2 values, which may reflect more active inflammatory processes in the early phase of the disease.

## Strengths and limitations

Our study's strengths include its prospective design, comprehensive imaging assessments, and consideration of key confounders. Additionally, by excluding patients with confirmed coronary artery disease, we were able to directly observe the myocardial changes connected to RA disease activity, independently of ischemic heart disease. Considering the limitations of our study, including the sample size, although adequate, limits the generalizability of our results. Limitations include the sample size, which, although adequate, may limit generalizability. The cross-sectional design also precludes causal inference. While DAS28-CRP values indicated heterogeneous disease activity, subgroup analysis was not feasible due to moderate and uneven group sizes. Nearly half of the RA cohort had hypertension, while none of the controls did; despite statistical adjustment, this discrepancy may have introduced residual confounding.

Finally, although many patients were receiving immunomodulatory therapy—including biologics, JAK inhibitors, NSAIDs, or glucocorticoids—we were unable to assess treatment-specific effects due to sample size constraints. Future studies should evaluate myocardial changes across disease activity levels and treatment exposures in larger, stratified cohorts.

## Conclusion

Our study has contributed to a better understanding of the CV consequences of RA and could help inform clinical decision-making and risk stratification in RA patients. Early identification and management of CV consequences of RA, strict control of CV risk factors, along with optimal control of the disease itself, are the cornerstones of diminishing CV morbidity and mortality.

## Data Availability

Data are available upon reasonable request to the corresponding author.

## Supplementary material

Supplementary Figure S1 Comparison between the myocardial deformation of RA patients pre- and post-adenosine. Comparison of myocardial deformation in RA patients before and after adenosine stress perfusion. The analysis includes left ventricular longitudinal and circumferential strain changes, indicating increased myocardial deformation under stress conditions. MD, myocardial deformation; GLS, global longitudinal strain; GCS, global circumferential strain.
