# Supplementary figures and images for: Subclinical myocardial changes in rheumatoid arthritis: cardiovascular magnetic resonance evidence of immuno-inflammatory remodeling
Source: Front Cardiovasc Med. 2025 Aug 21;12:1607018. doi: 10.3389/fcvm.2025.1607018 (PMC12408490; doi:10.3389/fcvm.2025.1607018)

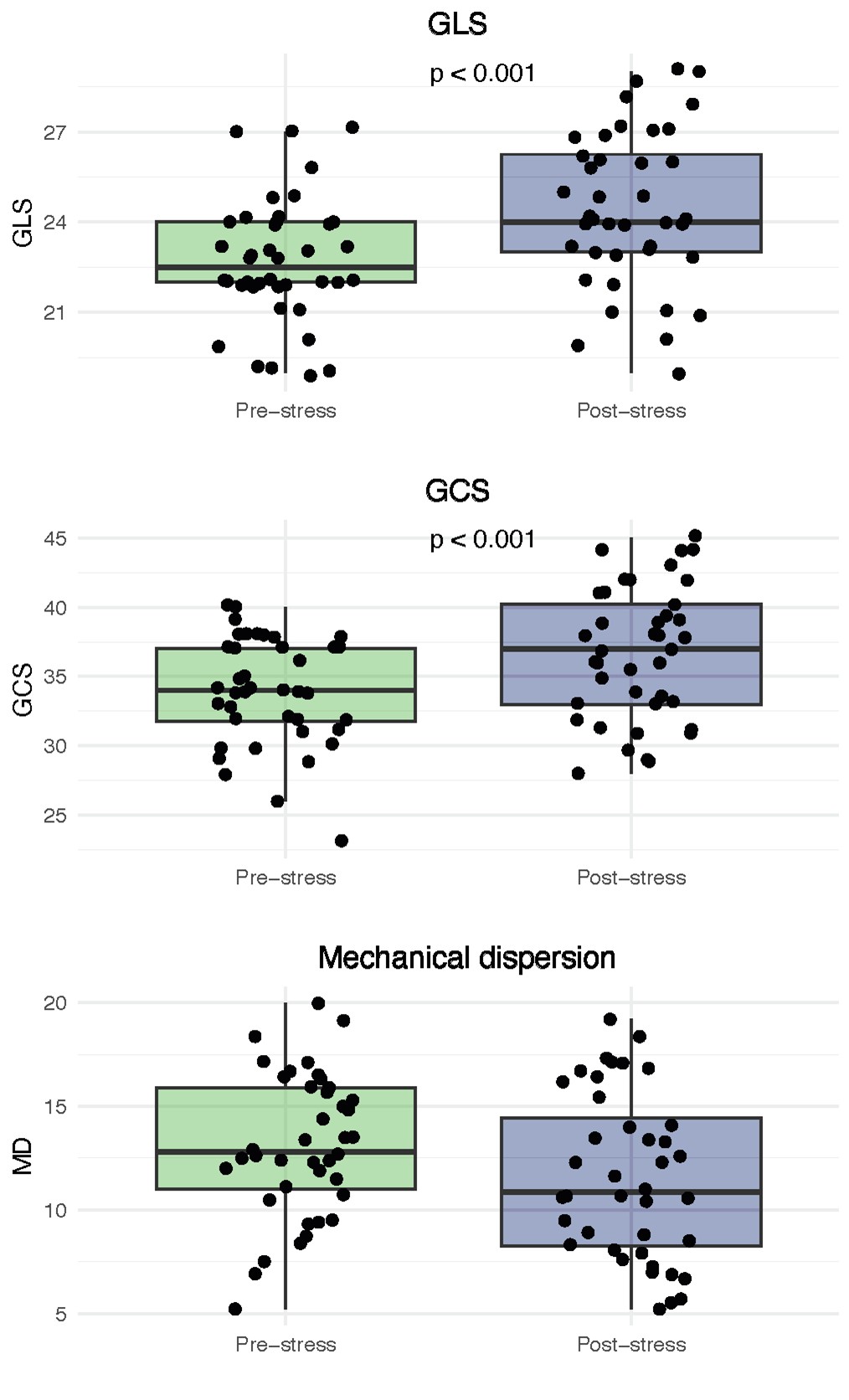

Supplement: Supplementary Figure S1 — Comparison between the myocardial deformation of RA patients pre- and post-adenosine. Comparison of myocardial deformation in RA patients before and after adenosine stress perfusion. The analysis includes left ventricular longitudinal and circumferential strain changes, indicating increased myocardial deformation under stress conditions. MD, myocardial deformation; GLS, global longitudinal strain; GCS, global circumferential strain. [file Image1.jpeg]
